# Top topics in HCV research arena

**DOI:** 10.1186/1471-2334-12-S2-S7

**Published:** 2012-11-12

**Authors:** Massimo Puoti, Roberto Rossotti, Giovanna Travi, Maurizio Orso, Maria Cristina Moioli

**Affiliations:** 1 Infectious Diseases Unit AO Ospedale Niguarda Ca’ Granda, 20162 Milano, Italy

## Abstract

A significant improvement in the rate of eradication of Hepatitis C Virus Genotype 1 has been achieved with the addition of Boceprevir and Telaprevir to pegylated interferon and ribavirin. These two drugs are the heralds of a new wave of antivirals that will improve the efficacy of pegylated interferon or even will substitute this drug in interferon free combinations. The results of phase II studies in patients naïve to treatment seem to be very promising strongly supporting the possibility of a large success for a first line all oral antiviral combination in interferon naïve. However, data observed in interferon experienced patients are less exciting and probably more complex treatment regimens will be needed to treat this patients’ population.

## Introduction

In the last 2 years the development of Directly Acting Antivirals (DAA) for the eradication of Hepatitis C Virus (HCV) infection and the availability of two Protease Inhibitors (PI) -boceprevir and telaprevir- have completely changed the scenario of HCV research.

Many interesting aspects of HCV epidemiology and pathogenesis, the relationship among HCV and diabetes or cardiovascular diseases, the improvements in the knowledge of immune response to HCV and even the research on potentially preventive or therapeutic vaccines have been obscured by the new exciting data on antivirals acting on HCV.

## Anti HCV DAA classification

Actually there are 4 classes of drugs on development with two targets: protease inhibitors, nucleoside/nucleotide polymerase inhibitors and non nucleosides polymerase inhibitors acting on the product of the Non Structural 5b (NS5b) gene of the polymerase (NS5b PolI), inhibitors of the Non Structural 5a (NS5a) gene of the polymerase (NS5aPolI), and cyclophillin inhibitors acting on an host protein that links viral polymerase.

## Development of anti HCV DAA

Currently approved are 2 protease inhibitors, telaprevir and boceprevir. In phase 3 trials now are 4 drugs: 2 HCV protease inhibitors TMC435 & BI1335; nucleotide GS7977; and NS5A BMS052. Phase 3 studies for these drugs should be finished in about one year with varying finish timelines between these drugs.

So in about 1 year we will have 2 brand new classes of drugs BMS052 the potent NS5A polymerase inhibitor, GS7977 the potent nucleotide polymerase inhibitor, plus the 2 new proteases currently in phase 3 BI1335 & TMC435. At this time we don't know if clinicians will be able to combine the NS5A+GS7977 or a 3-drug combination of a protease TMC435 or BI1335 + the NS5A BMS052+GS7977 [1].

## DAA efficacy in phase II studies

**Figure 1:**
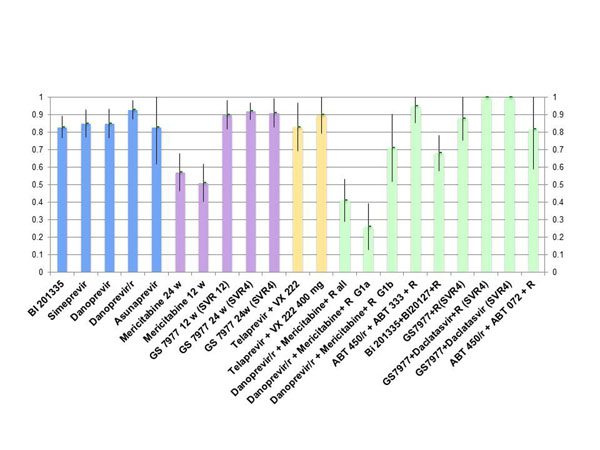
**Phase II trials: SVR rate and 95% CI with DAA in HCVG1 naives.** Blue, Triple combo with Protease Inhibitors (PI) + Pegylated interferon (P) + Ribavirin ( R ). Purple, Triple combo with NS5aPol inhbitors + PR. Yellow, QUAD two antivirals acting on viral protease and polymerase with pegylated interferon + ribavirin. Green, Interferon free combos. They are NOT HEAD TO HEAD STUDIES.

In naïve patients phase II studies with new protease inhibitors or with polymerase inhibitors administered in combination with pegylated interferon + ribavirin showed Sustained Virologic Response (SVR) rates higher than 80% and in some cases higher than 90% with shorter treatment duration, less side effects, more convenient schedules in comparison with triple therapy regimen conducted with boceprevir or telaprevir. However the same efficacy was observed with some interferon free combinations of protease inhibitors or polymerase inhibitors with or without ribavirin. Results obtained in phase II studies presented at EASL in 2012 [2-14] are summarized in Figure 1.

In patients with HCV genotype 2 or 3 these results have also been confirmed with nucleotide polymerase inhibitors combined with ribavirin which achieved 100% SVR rates in small pilot studies [7,14] (Table 1). So it could be supposed that in the future interferon free combination of antivirals with or without ribavirin will be the first line in the treatment of naïve patients with HCV.

**Figure 2:**
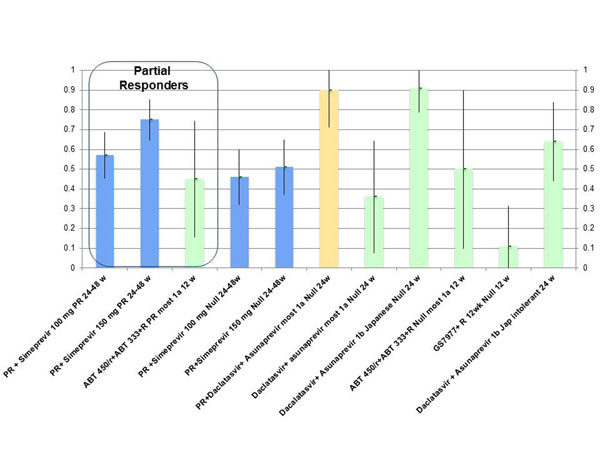
**Phase II trials: SVR rate with DAA in HCVG1 non responders.** Blue, Triple combo with PI + PR. Yellow, QUAD with P+R. Green, interferon free combos.

In experienced patients with a null or partial response to a previous course of pegylated interferon and ribavirin the results of interferon free combinations are still less impressive; they are good in patients infected with HCV G1b but the rate of SVR in patients with HCV Genotype 1a infection seems to be suboptimal. Results obtained in phase II studies in experience patients with HCV G1 infection presented at EASL are summarized in Figure 2.

**Table 1:** Phase II trials: SVR Rate with DAA in HCVG 2-3 naives and experienced (NOT HEAD TO HEAD STUDIES)

DAA	Class	DAA Duration	PR Duration	N	SVR 4*/24 Naives	SVR 4 experienced
PR+GS-7977	NS5bPolI	12 wk 12 wk 12 wk	12 wk 8 wk 4 wk	11 10 9	11/11 10/10 10/10	

GS-7977 + R	Ns5bPolI	12 wk	0	11	11/11	

GS-7977+ R	Ns5bPolI	12 wk	0	25		12/15

GS7977 + Daclatasvir + R	NNS5bPolI + NS5aPolI + R	24 wk 24 wk	0 0	30 14	28/30* 11/14*	

Abbreviations: DAA: Directly Acting Antiviral; NS5b PolI : NS5b polymerase inhibitor; NS5a Polymerase Inhibitor; PR Pegylated Interferon combined with Ribavirin; wk: week; SVR: Sustained Virologic Response; SVR 4: HCVRNA below the level of detection with Roche TaqMan assay 4 weeks after treatment withdrawal; SVR 24: HCVRNA below the level of detection with Roche TaqMan assay 24 weeks after treatment withdrawal

Thus three or four antivirals in combination or combinations of antivirals with pegylated interferon will probably be necessary to treat the most difficult to treat patients: those with HCV G1a infection and/or poor response to pegylated interferon and ribavirin and/or with advanced fibrosis.

## Safety data with new DAA

One of the major challenges given by adding telaprevir or boceprevir to pegylated interferon combined with ribavirin is the addition of the toxicities given by these drugs to the engaging series of side effects given by the standard of care. Rash, anemia, neutropenia, thrombocytopenia, dysgeusia, anorectal discomfort have been found more frequently in patients treated with boceprevir and telaprevir. So the lack or the reduction of side effects is a pre requisite for the development of new anti HCV DAA. In fact, most of the new DAA have showed a better tolerability profile in phase II studies that became quite optimal in interferon free combinations. Sometimes phase II studies are sufficient to reveal important side effects and it is demonstrated by the fact that the development of several antivirals has been stopped because of occurrence of severe side effects in few patients. Nevertheless there is a strong need for large sample size in order to really assess the safety profile of these drugs. Some safety signals observed in dose finding studies with new drugs (i.e., rash with BI 201335, hypertransaminasemia with unboosted danoprevir) suggest the need for a careful evaluation of side effects in phase III studies. Other side effects such as gastrointestinal symptoms or jaundice due to Gilbert-like hyperbilirubinemia are less important for motivated patients enrolled in clinical phase II trials, but could reduce the acceptability of and adherence to treatment in the real life setting.

## Caveats in the results of phase II studies on anti HCV DAA

Even if the results of these studies are exciting there are some caveats that should be taken into account:

• Patients with HCV infection even if stratified according to genotype are very heterogeneous;

• In most of the studies and especially with interferon-free combinations the sample size was extremely small, so they should be regarded as proof of concept studies rather than dose finding studies, as are most of the traditional phase II studies;

• The duration of antiviral therapy in most studies was 12 weeks: obtaining an eradication of HCV with only three months of treatment is exciting but is not the Holy Grail and longer treatment duration could also be acceptable in the most difficult to treat patients. So data with longer treatment duration are still needed in harder to treat population;

• In some studies a negative HCV RNA 4 weeks after stopping treatment was assumed as a proof of HCV eradication, but probably we still have to identify the optimal follow up duration needed to define SVR to Interferon-free regimens;

• Adherence and resistance are crucial for interferon-free combinations and when prescribing them first-line treatments, a second line rescue line without cross-resistance to the drugs previously administrated should be identified.

## Conclusions

In summary, exciting new times are coming for HCV treating physicians and persons living with HCV. However we have to be prepared to face an insidious and able enemy who should probably be eradicated only combining prudence and knowledge of its heterogeneous nature.

## Competing interests

Massimo Puoti has received fees as a member of occasional Advisory Boards, and/or speaker in own events and/or teacher in courses for employees and/or research grants and/or is involved as principal investigator in trials supported from the following drug companies: Abbott, BMS, Boheringer Ingelheim, Gilead Sciences, Janssen, MSD, Roche, Vertex.

Roberto Rossotti, Giovanna Travi, Maurizio Orso, and Maria Cristina Moioli have been involved as coinvestigators in trials supported by Abbott, BMS, Boheringer Ingelheim, Gilead Sciences, Janssen, Roche.

## List of abbreviations used

DAA: Directly Acting Antivirals; HCV: Hepatitis C Virus; PI: Protease Inhibitors; SVR: Sustained Virologic Response; PI: Protease Inhibitor; NS5b PolI: NS5b polymerase inhibitor; NS5a PolI: Polymerase Inhibitor; PR: Pegylated Interferon combined with Ribavirin; wk: week; SVR 4: HCVRNA below the level of detection with Roche TaqMan assay 4 weeks after treatment withdrawal; SVR 24: HCVRNA below the level of detection with Roche TaqMan assay 24 weeks after treatment withdrawal.

## Declarations

Publication of this supplement was partly supported by an unrestricted grant provided by Roche. The articles were independently prepared by the authors with no input from Roche. Roche were not involved in selecting the articles for the supplement. The pegylated interferon treatment mentioned in this article is produced by Roche.
